# Curcumin Analog CH-5 Suppresses the Proliferation, Migration, and Invasion of the Human Gastric Cancer Cell Line HGC-27

**DOI:** 10.3390/molecules23020279

**Published:** 2018-01-30

**Authors:** Gabriel Silva, Felipe Teixeira Lima, Viviane Seba, Ana Laura Mendes Lourenço, Thaise Graminha Lucas, Bianca Vieira de Andrade, Guilherme Silva Torrezan, Carlos Roberto Polaquini, Marcelo Engracia Garcia, Lucélio Bernardes Couto, Reinaldo Bulgarelli Bestetti, Suzelei de Castro França, Ana Lúcia Fachin, Luis Octavio Regasini, Mozart Marins

**Affiliations:** 1 Biotechnology Unit, University of Ribeirão Preto, Av. Costábile Romano, 2201, Ribeirão Preto, SP CEP 14096-900, Brazil; biel-189@hotmail.com (G.S.); lima.ft@hotmail.com (F.T.L.); vivianeseba@gmail.com (V.S.); analaura.mendes@hotmail.com (A.L.M.L.); thaisegl.uru@hotmail.com (T.G.L.); bandrade@hotmail.com (B.V.d.A.); sfranca@unaerp.br (S.d.C.F.); afachin@unaerp.br (A.L.F.); 2 Medicine School, University of Ribeirão Preto, Av. Costábile Romano, 2201, Ribeirão Preto, SP CEP 14096-900, Brazil; mgarcia@unaerp.br (M.E.G.); lubercouto@gmail.com (L.B.C.); rbestetti@unaerp.br (R.B.B.); 3 Laboratory of Green and Medicinal Chemistry, Department of Chemistry and Environmental Sciences, Institute of Biosciences, Humanities and Exact Sciences, São Paulo State University (UNESP), São José do Rio Preto, SP CEP 15054-000, Brazil; guilherme_panz@hotmail.com (G.S.T.); carlos_polaquini@hotmail.com (C.R.P.)

**Keywords:** gastric cancer, curcumin, matrix metalloproteinase 2, migration, invasion, apoptosis

## Abstract

Gastric cancer is one of the most frequent malignant tumors in the world. The majority of patients are diagnosed with metastatic gastric cancer, which has a low survival rate. These data reinforce the importance of studying the anticancer activity of new molecules with the potential to suppress gastric cancer metastasis. Curcumin is a well-studied compound that has demonstrated anti-metastatic effects. Here we investigated if CH-5, a curcumin derivative compound, has anti-metastatic properties in the human gastric cancer cell line HGC-27. Firstly, we found that CH-5 decreased viability and induced apoptosis in HGC-27 cells in a dose-dependent manner. Additionally, CH-5 suppressed the migration and invasion of HGC-27 cells by downregulating the expression and collagenase activity of matrix metalloproteinase 2 in a dose-dependent manner. In conclusion, CH-5 showed anticancer activities, including the induction of apoptosis, and the suppression of migration and invasion in HGC-27 cells, suggesting that CH-5 can be a lead molecule for the development of anti-metastatic drugs for gastric cancer therapy.

## 1. Introduction

Gastric cancer (GC) is one of the most commonly diagnosed types of malignant tumors worldwide and it is the third leading cause of cancer-related deaths. In humans, GC can be connected to life habits, viral infection, and genetic or environmental factors [1]. The survival of people with GC is still low, with only about 24% exhibiting a five-year survival rate. This high mortality is due to late diagnosis, often when the cancer has already metastasized [2]. Based on this, studies related to the genetics of GC, new therapeutic targets, and drugs are needed to change this scenario.

The degradation of the extracellular matrix (ECM) is an important mechanism for the maintenance and renewal of tissues in general. On the other hand, the deregulation of this process plays a role in the development of some types of cancer, such as gastric cancer [3]. There are several proteins involved in the degradation of the ECM; however, the matrix metalloproteinases (MMPs) are considered the most important [4].

Metalloproteinases are zinc-dependent endopeptidases that can degrade all components of the ECM [4]. Based on structure and function, MMPs can be divided into: collagenases 1, 2, 3 (MMP-1, -8 and -13, respectively), stromelysins 1 and 2 (MMP-3 and -10), gelatinases (MMP-2 and -9), and membrane-type MMPs (MT-MMP), respectively MMP-14, -15, -16, -17, -24, and -25 [4,5]. Matrix metallopeptidase 2 (MMP-2), which is classified as type IV collagenase, is one of the most important metalloproteinases because it can degrade different types of collagens. In addition, MMP-2 is the most expressed MMPs and it is found in the vast majority of tissues and cells [6,7]. MMP-2 plays a crucial role in the migration and invasion of cancer cells. Through the degradation of basement membrane proteins (specialized ECM), this MMP can facilitate stromal and vascular invasion by tumor cells, thus allowing these cells to spread to distant sites from the primary tumor [8,9]. Some studies report the high expression of MMP-2 in several types of cancer, among them the GC, and for that reason the inhibition of this metalloproteinase becomes a potential attempt to control tumor progression and metastasis [10,11].

Natural small molecules could be significant therapeutic agents for the treatment of numerous diseases, including cancer. Among these molecules, curcumin, a phenolic compound obtained from the rhizomes of *Curcuma longa* L., has been widely studied and has shown antioxidant, anti-inflammatory, and anticancer activity [12]. Furthermore, curcumin has been demonstrated to inhibit cancer progression by suppressing MMP-2 activity [13,14,15]. However, curcumin shows poor solubility that results in low bioavailability. A variety of experimental studies based on cells and animals have shown that structural modifications in the β-diketone moiety, the aromatic rings, or the flanking double bonds conjugated to the β-diketone moiety of curcumin can improve its bioavailability, besides enhancing the anticancer activity [16]. Our research group has been screening monoketone analogs of curcumin and selected CH-5 (4,4′-[(2-Oxo-1,3-cyclohexanediylidene)-di(*E*)methylylidene]dibenzonitrile) as an interesting compound to be tested for anticancer activity in different tumor cells [17,18]. In the present study, we demonstrated that CH-5 suppresses cell migration and invasion accompanied by the inhibition of MMP-2 in the human gastric cell line HGC-27.

## 2. Results

### 2.1. CH-5 Reduces Cell Viability and Induces Apoptosis in a Gastric Cancer Cell Line

**Figure 1:**
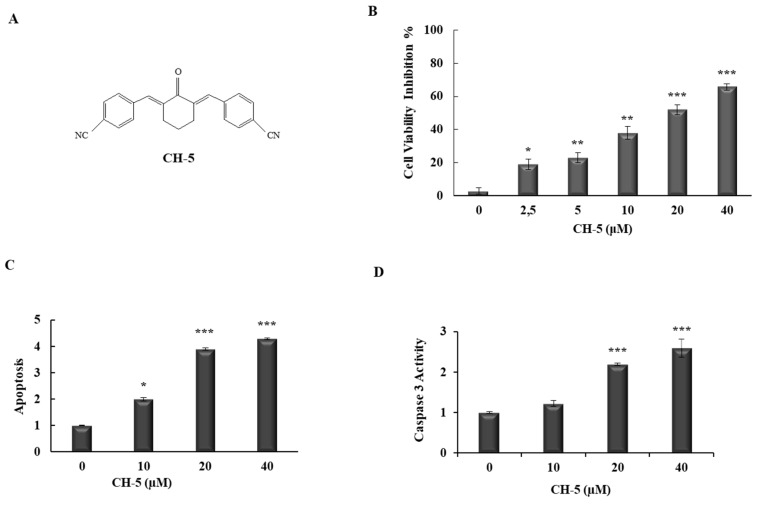
Cellular viability of HGC-27 cells treated with CH-5. (**A**) Chemical structure of CH-5; (**B**) HGC-27 cells were treated with DMSO (0.1%) or with 0, 2.5, 5, 10, 20, 40 µM of CH-5, and after 24 h their viability was measured by an MTT assay. Each experiment was performed in quadruplicate; (**C**) Apoptosis induction by CH-5. HGC-27 cells were treated with DMSO (0.1%), or 10, 20, and 50 µM of CH-5, and after 24 h the rate of apoptotic cells was assessed by double staining (Annexin V-FITC/PI) flow cytometry assay. Y-axis represents fold induction compared to DMSO-treated cells; (**D**) Apoptosis was further confirmed by measuring caspase 3 activity in HGC-27 cells treated with CH-5. After treatment with CH-5 at the indicated concentrations, protein lysates were mixed with caspase-3 substrate, and then caspase-3 activity was determined by measuring absorbance, according to the manufacturer’s instructions described in the materials and methods section. Y-axis represents relative caspase activity compared to DMSO-treated cells. In all experiments, the data represent the mean ± SD of three experiments. * *p* ≤ 0.05, ** *p* ≤ 0.01, and *** *p* ≤ 0.001 compared with DMSO control.

To determine the effects of CH-5 (Figure 1A) on gastric cancer cell viability, we treated HGC-27 cells with CH-5 at different doses for 24 h. The MTT results showed that CH-5 reduced the viability of HGC-27 cells in a dose-dependent manner with significant inhibition (51%) at 20 µM (Figure 1B). To verify whether the reduction of HCG-27 cell viability was correlated to the induction of apoptosis, we performed Annexin V-FITC/PI double staining using flow cytometry. In conformity with the inhibition of cell viability, the CH-5 also increased apoptosis in a dose-dependent manner; the fold induction of apoptosis was 2.0, 3.9, and 4.3 in HGC-27 cells treated with CH-5 at 10, 20, and 40 µM, respectively (Figure 1C). Caspase 3 activity is also a biomarker of apoptosis and, as shown in Figure 1D, the treatment of HGC-27 cells with increasing concentrations of CH-5 resulted in augmented levels of caspase 3 activity. The data indicated that CH-5 has an antiproliferative effect on gastric cancer cells mediated by apoptosis induction.

### 2.2. CH-5 Decreases Migration and Invasion in a Gastric Cancer Cell Line

**Figure 2:**
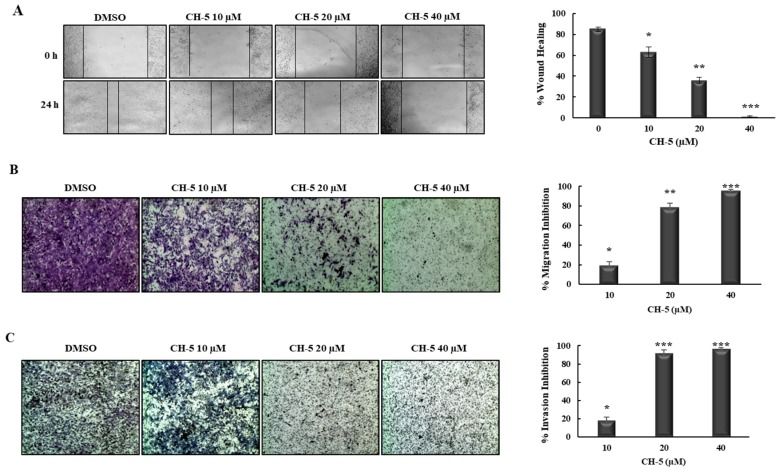
CH-5 inhibited cell migration and invasion in HGC-27 cells. (**A**) The effect of CH-5 on HGC-27 cell migration was evaluated by a wound healing assay. HGC-27 cells were scratched and treated with 0, 10, 20, and 40 μM of CH-5 for 0 and 24 h. The migration was observed under a phase-contrast microscope at a magnification of 40×. Migration inhibition (%) after treatment with CH-5 was calculated, and quantitative results are illustrated in the right panel; (**B**) The inhibitory effect of CH-5 on HGC-27 cell migration was detected by a Transwell assay. Cells in serum-free medium were plated onto the upper chamber of the Transwell. Complete medium (10% serum) containing CH-5 at the indicated doses was added to the lower chamber. After 24 h, cells on the bottom side of the Transwell membrane were stained and observed by manual counting and measuring absorbance at 490 nm. Migration Inhibition (%) was calculated and quantitative results are illustrated in the right panel; (**C**) For the invasion assay, the Transwell membrane was pre-coated with Matrigel, following which the cells were plated and treated as described above. Invasion Inhibition (%) was calculated and quantitative results are illustrated in the right panel. In all experiments, the data represent the mean ± SD of three experiments. * *p* ≤ 0.05, ** *p* ≤ 0.01, and *** *p* ≤ 0.001 vs. DMSO group.

We evaluated the effect of CH-5 on HCG-27 cell migration and invasion using a wound-healing assay and a Transwell assay. The wound-healing assay showed that CH-5 significantly inhibited wound closure in a concentration-dependent manner (Figure 2A). In order to confirm the cell migration inhibition mediated by CH-5, we performed a Transwell assay. In agreement with the wound-healing assay, as shown in Figure 2B, the treatment with CH-5 for 24 h dramatically reduced the number of HGC-27 cells that had migrated through the Transwell membrane in a dose-dependent manner, with substantial inhibition (79.8%) at 20 µM. To evaluate the effect of CH-5 on cell invasive capacity, we used Transwell coated with Matrigel, as shown in Figure 2C; a number of HGC-27 cells that had invaded the Matrigel was significantly decreased (92%) by CH-5 at 20 µM.

### 2.3. CH-5 Reduces the Transcriptional Levels and Activity of MMP-2

**Figure 3:**
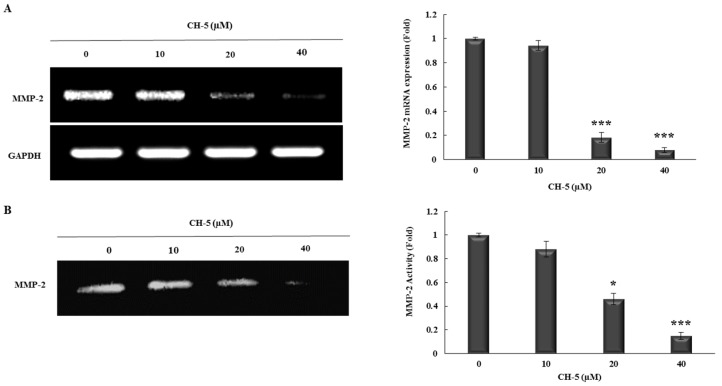
CH-5 decreased transcriptional levels and protease activity of MMP-2 in HGC-27 cells. (**A**) HGC-27 cells were treated with 0, 10, or 40 µM of CH-5; after 24 h the total RNA was isolated and then subjected to conventional RT-PCR using primers for MMP-2 and housekeeping gene GAPDH. A representative gel (left) and densitometry analysis from three independent experiments (right) are shown; (**B**) HGC-27 cells were treated with CH-5 (0, 10, 20, and 40 µM) for 24 h and then the activity of secreted MMP-2 was measured by gelatin zymography assay. A demonstrative gel (left) and quantitative results (right) from three independent experiments are presented. In all experiments, * *p* ≤ 0.05 and *** *p* ≤ 0.001 vs. DMSO-treated cells.

HGC-27 cells were treated for 24 h with 0, 10, 20, and 40 µM of CH-5, and then the transcripts of MMP-2 were measured by conventional RT-PCR. We found that mRNA levels of MMP-2 were decreased by CH-5 in a dose-dependent manner with a significant reduction (fold—5.5) at 20 µM (Figure 3A). To evaluate whether MMP-2 downregulation at the transcription level by CH-5 affects its activity, we performed a Gelatin zymography assay. HGC-27 cells were treated for 24 h with indicated doses of CH-5, and then the activity of MMP-2 was assessed. CH-5 treatment reduced the MMP-2 collagenase activity in a dose-dependent manner with a remarkable effect at 20 µM (fold—2.3) (Figure 3B).

## 3. Discussion

Metastasis is a complex process involving various steps: detachment, invasion, intravasion, migration to distant sites through the circulatory system, extravasation, and proliferation of cancer cells in competent organs. Initially, metastatic cells need to detach from the primary tumor, migrate and invade the basement membrane, then enter and travel into the blood and lymph vessels, leave the vascular system, and lastly adhere and grow at a distant site [19,20]. Metalloproteinases, through the degradation of the extracellular matrix, play a crucial role in all these steps; consequently, a common strategy of tumor cells to trigger metastasis is the overexpression of metalloproteinases, mostly MMP-2 [17,18].

Based on this, target MMP-2 expression in cancer cells is a reasonable strategy for decreasing metastasis, and the use of dietary compounds, in particular curcumin, has gained attention [13,14,15,21,22]. We have studied the anticancer effects of CH-5, a curcumin derivative, which has been shown in our studies to have better potential anticancer activities than curcumin.

In the present study, we investigated whether CH-5 has antiproliferative and pro-apoptotic properties and, finally, whether CH-5 inhibits migration and invasion in gastric cancer cells by reducing the expression of MMP-2.

In order to clarify the cytotoxic effect of CH-5 on a gastric cancer cell line (HGC-27), an MTT assay was performed. We found that CH-5 significantly suppressed cell viability in a dose-dependent manner in HGC-27 cells. To elucidate whether the suppression of HGC-27 cell viability was associated with the induction of apoptosis, we used flow cytometry analysis. In agreement with the reduction of cell viability, treatment with CH-5 increased apoptosis in a dose-dependent manner. We have found in other cell lines that CH-5 is capable of inducing 3/7 caspase activity and then promoting the cleavage of PARP [17,18], and curcumin has already been shown to induce caspases-mediated apoptosis in gastric cancer [23,24]. Thus, CH-5 is likely to trigger apoptosis in gastric cancer cells by the induction of caspases.

We further used wound-healing and Transwell assays to investigate the effect of CH-5 on cell migration and invasion. As it has been demonstrated in vitro and in vivo, curcumin suppresses the migration and invasion of cancer cells [15,25]. Here, we observed that CH-5 also suppresses migration and invasion in gastric cancer cell line HGC-27 in a dose-dependent manner. Interestingly, at the same dose, CH-5 inhibited migration and invasion more strongly than cell viability (Figure 1B and Figure 2B), suggesting that the anti-metastatic effect of CH-5 is not only linked to cytotoxicity.

MMP-2 is a critical protease for tumor cell migration and invasion by degrading the extracellular matrix. Overexpression of MMP-2 has been associated with increased tumor metastasis and worse prognosis in several cancers, including gastric cancer [10,11]. Building on this knowledge, we evaluated whether the inhibition of migration and invasion mediated by CH-5 was associated with the alteration of MMP-2 activity. First, through conventional RT-PCR analysis, we found that CH-5 can downregulate MMP-2 gene expression. Next, using a zymography assay, we observed that CH-5 also decreased MMP-2 collagenase activity. It has been shown that the inhibition of MMP-2 expression appears to be connected to the reduction of transcription factor Sp1 [26,27], and we have found in other cancer cell lines that CH-5 decreases the levels of Sp1 protein [17,18]; so probably the anti-migration and anti-invasion effects of CH-5, at least in part, can be related to the downregulation of the Sp1/MMP-2 axis.

In this study, we found that CH-5, a curcumin-related compound, reduces cell viability by the induction of apoptosis in gastric cancer cell line. Furthermore, CH-5 decreases the expression and activity of MMP-2 protein, which plays a critical role in cancer cell migration and invasion. The data presented points toward further studies on the potential anti-metastatic activity of CH-5.

## 4. Materials and Methods

### 4.1. Cell Culture and Chemicals

The human gastric cancer cell line HGC-27 was purchased from the Rio de Janeiro Cell Bank (BCRJ, Federal University of Rio de Janeiro, Rio de Janeiro, Brazil). HGC-27 cells were grown in Dulbecco’s Modified Eagle Medium (Sigma-Aldrich^®^, St. Louis, MO, USA) supplemented with 10% fetal bovine serum (FBS), 100 U/mL penicillin, and 100 µg/mL streptomycin (Sigma-Aldrich^®^). Cells were cultured at 37 °C under a humidified atmosphere of 5% CO_2_ for all experiments. CH-5, a curcumin analog (Figure 1A), was provided by Dr. Luis Octávio Regasini (Department of Chemistry and Environmental Chemistry, São Paulo State University, São Paulo, Brazil).

### 4.2. Cell Viability Assay

The cell viability of HGC-27 cell was measured by an MTT assay. In brief, cells were seeded in 96-well plates at a density of 5 × 10^3^ cells/well. After an overnight culture, cells were treated in quadruplicate with 0, 2.5, 5, 10, 20, or 40 µM of CH-5 for 24 h. DMSO was used as a solvent at a final concentration of 0.1%, which is considered atoxic for the cells. After treatment, the medium was replaced with fresh medium; 20 µL MTT solution (5 mg/mL) was added to each well, and the plates were incubated for an additional 3 h. The absorbance was then measured spectrophotometrically at a wavelength of 550 nm by a microplate reader (MultiSkan FC, Thermo Scientific, Waltham, MA, USA). The results were plotted as the percentage of inhibition of cell viability (ICV) calculated as follows: ICV (%) = [1 − (absorbance of treated group/absorbance of control group)] × 100. Data represent the average ± SD from three independent experiments combined.

### 4.3. Apoptosis Assay

Apoptosis analysis was performed with double labeling (Annexin-V/propidium iodide), using an FITC Annexin V Apoptosis Detection Kit I (BD Biosciences), according to the manufacturer’s recommendations, with slight modifications. HGC-27 cells were grown in a 60-mm dish to 80% confluence, then treated with 10, 20, or 40 µM of CH-5 in a medium with 10% FBS for 24 h. After this treatment, adherent and floating cells were collected by centrifugation, washed with PBS, and resuspended in 300 µL of 1× binding buffer. Next, 5 µL of FITC Annexin V and 5 µL of propidium iodide were added and the cells were incubated at room temperature in the dark for 15 min. Finally, cells were analyzed using a BD FACSCalibur™ Flow Cytometer (BD Biosciences, San Jose, CA, USA).

### 4.4. Caspase 3 Assay

The activity of caspase 3 was assessed using a caspase 3 colorimetric assay (Sigma-Aldrich^®^). The HGC-27 cells cultured in 60-mm plates were treated with 10, 20, or 40 µM of CH-5 in a medium with 10% FBS for 24 h. Next, protein lysates were obtained in 1× lysis buffer. Then, 30 µg of total proteins (in 10 µL) were distributed into 96-well plates and mixed with 80 µL of 1× assay buffer and 10 µL of caspase-3 substrate. After incubation at 37 °C in the dark for 3 h, the absorbance was measured at 405 nm by a microplate reader (MultiSkan FC).

### 4.5. Wound-Healing Assay

A wound-healing assay was performed to analyze cell migration capacity according to the published protocols of literature with minor modifications [28]. Briefly, HCG-27 cells were grown in 24-wells plate until the cell monolayer reached nearly 100% confluency, then a wound was created with a sterile 10-μL pipette tip. Floating cells were removed, and attached cells were exposed to the indicated concentration of CH-5 for 0 and 24 h. After the incubation time, the scratched areas were photographed in an optical microscope. The wound area was measured using ImageJ software (ImageJ2, National Institutes of Health, Bethesda, MD, USA). Migration rates of CH-5 group and DMSO group were calculated as follows: Migration rate (%) = [(wound area at 0 h − wound area at 24 h)/wound area at 0 h] × 100%.

### 4.6. Transwell Assay

A Transwell cell assay was used to analyze the effect of CH-5 on the migration and invasion capacity of HCG-27 cells, according to the published protocols of literature with minor modifications [29]. Briefly, cells were serum-starved overnight, and then 2 × 10^5^ cells resuspended in 200 µL serum-free medium were seeded into the upper chamber of a 24-well Transwell plate with an 8 µm pore size (Corning, Corning, NY, USA). Into the lower chamber, 750 µL of complete medium (with 10% serum) containing different concentrations of CH-5 was added. After treatment for 24 h, the migrated cells attached on the bottom side of the Transwell membrane were fixed with 3.7% of paraformaldehyde for 2 min, permeabilized with methanol 100%, and stained with Wright’s Giemsa solution for 15 min at room temperature. For the invasion assay, the membrane of the Transwell was pre-coated with 0.75 mm of Matrigel (Corning^®^) according to the manufacturer’s instructions, and then the same step detailed for the migration assay was performed. Either for migration and invasion, the cells were destained with 100 µL of cells of 33% acetic acid. The destaining solution was collected and the absorbance was measured at a wavelength of 490 nm by a microplate reader (MultiSkan FC). The inhibition of migration or invasion was calculated as follows: Inhibition (%) = [1 − (absorbance of treated group/absorbance of control group)] × 100. Data represent the average ± SD from three independent experiments.

### 4.7. Reverse Transcription-Polymerase Chain Reaction (RT-PCR)

Conventional RT-PCR was performed to analyze the matrix metalloproteinase-2 (MMP-2) expression. In short, HCG-27 cells were seeded in 6-well plates at a density of 5 × 10^5^ cells/well and cultured overnight. Next, the cells were treated with 0, 10, 20, or 40 µL of CH-5 in serum-free medium for 24 h. After treatment, total RNA was isolated using Illustra™ RNASpin Mini Kit (GE Healthcare, Little Chalfont, UK) and treated with DNase I Amplification Grade (Sigma-Aldrich^®^). Then, 1 µg of RNA was used for the synthesis of cDNA using a High-Capacity cDNA Reverse Transcription kit (Applied Biosystems). PCR was carried out using RedTaq Mix (Sigma-Aldrich^®^) with human primers as follows: MMP-2 Fwd 5′-ttccccttcttgttcaatgg-3′ and Rev 5′-atttgttgcccaggaaagtg-3′; and GAPDH Fwd 5′-gaccacagtccatgccatcact-3′ and Rev 5′-tccaccaccctgttgctgtag-3′. The thermal cycling conditions were as follows: initial denaturation at 94 °C for 2 min, followed by 26 cycles of 94 °C for 30 s, 60 °C for 30 s and 72 °C for 30 s, and a final extension at 72 °C for 5 min. The PCR products were electrophoresed on 1.5% agarose gel and photographed under UV light. The intensity of bands was analyzed by densitometry using the GAPDH band (constitutive expressing gene) as a reference.

### 4.8. Gelatin Zymography

Gelatin zymography was performed to quantify the matrix metalloproteinase-2 (MMP-2) activity. Briefly, HCG-27 cells were cultured in 6-well plates (5 × 10^5^ cells/well) followed by treatment with 0, 10, 20, or 40 µL of CH-5 in serum-free medium for 24 h. After treatment, the supernatant (conditioned medium) for each sample was collected and then separated by 0.1% gelatin-7% SDS-PAGE electrophoresis. Next, the gels were washed in 2.5% Triton X-100 three times (30 min each) at room temperature and incubated in reaction buffer (10 mM CaCl_2_, 40 mM Tris-HCl, and 0.01% NaN_3_, pH 8.0) at 37 °C for 18 h. Gels were washed with distilled water, and stained with Coomassie brilliant blue R-250. After destaining steps, bands were digitally scanned, and the gelatinolytic activities were measured using ImageJ software.
